# Additive effects of two quantitative trait loci that confer *Rhopalosiphum maidis* (corn leaf aphid) resistance in maize inbred line Mo17

**DOI:** 10.1093/jxb/eru379

**Published:** 2014-09-23

**Authors:** Mariam Betsiashvili, Kevin R. Ahern, Georg Jander

**Affiliations:** ^1^Boyce Thompson Institute for Plant Research, Ithaca, New York, 14853, USA

**Keywords:** DIMBOA, *Rhopalosiphum maidis*, aphid, benzoxazinoid, callose, maize, quantitative trait.

## Abstract

Plants show considerable within-species variation in their resistance to insect herbivores. In the case of *Zea mays* (cultivated maize), *Rhopalosiphum maidis* (corn leaf aphids) produce approximately twenty times more progeny on inbred line B73 than on inbred line Mo17. Genetic mapping of this difference in maize aphid resistance identified quantitative trait loci (QTL) on chromosomes 4 and 6, with the Mo17 allele reducing aphid reproduction in each case. The chromosome 4 QTL mapping interval includes several genes involved in the biosynthesis of DIMBOA (2,4-dihydroxy-7-methoxy-1,4-benzoxazin-3-one), a maize defensive metabolite that also is required for callose accumulation in response to aphid feeding. Consistent with the known association of callose with plant defence against aphids, *R. maidis* reproduction on B73×Mo17 recombinant inbred lines was negatively correlated with both DIMBOA content and callose formation. Further genetic mapping, as well as experiments with near-isogenic lines, confirmed that the Mo17 allele causes increased DIMBOA accumulation relative to the B73 allele. The chromosome 6 aphid resistance QTL functions independently of DIMBOA accumulation and has an effect that is additive to that of the chromosome 4 QTL. Thus, at least two separate defence mechanisms account for the higher level of *R. maidis* resistance in Mo17 compared with B73.

## Summary

At least two genetic loci contribute to the high aphid resistance observed in the seedlings of maize inbred line Mo17. One of these loci increases the biosynthesis of defence-related benzoxazinoids.

## Introduction

Cultivated maize (*Zea mays*) is a genetically diverse crop plant that exhibits wide variation in its resistance to insect herbivores (McMullen *et al.*, 2009a; Meihls *et al.*, 2012). Genetic mapping has identified quantitative trait loci (QTL) that confer resistance to several maize-feeding insects, including *Diatraea grandiosella* (southwestern corn borer; Khairallah *et al.*, 1998), *Ostrinia nubilalis* (European corn borer; Papst *et al.*, 2004)*, Ostrinia furnacalis* (Asian corn borer; Xia *et al.*, 2010), *Helicoverpa zea* (corn earworm; Byrne *et al.*, 1998), *Spodoptera frugiperda* (fall armyworm; Brooks *et al.*, 2007), and *Sitophilus zeamais* (maize weevil, García-Lara *et al.*, 2009). Discovery of the actual genetic basis of such insect resistance QTL has been facilitated by the genome sequence of maize inbred line B73 (Schnable *et al.*, 2009), as well as by populations of recombinant inbred lines (RILs) and near-isogenic lines (NILs) that have been created from B73 and a diverse set of other maize inbred lines (Eichten *et al.*, 2011; Lee *et al.*, 2002; McMullen *et al.*, 2009b; Yu *et al.*, 2008).

The proximal causes of natural variation in maize insect resistance have been linked to the production of defensive proteins and secondary metabolites. Protease inhibitors (Tamayo *et al.*, 2000), cysteine proteases (Pechan *et al.*, 2000), and ribosome-inactivating proteins (Chuang *et al.*, 2014) provide protection against lepidopteran herbivory. Known maize defensive metabolites include chlorogenic acid (Cortés-Cruz *et al.*, 2003), maysin (Rector *et al.*, 2003), and benzoxazinoids (Frey *et al.*, 2009). Among these, the benzoxazinoids, which provide resistance to a large number of herbivores and pathogens (Niemeyer, 2009), have been studied the most extensively. The core biosynthetic pathway for 2,4-dihydroxy-7-methoxy-1,4-benzoxazin-3-one acid glucoside (DIMBOA-Glc) is encoded by eight genes (*Bx1*–*Bx8*) that are tightly linked at the top of maize chromosome 4. Two additional genes, *Igl1* and *Bx9*, which encode the same enzymatic functions as *Bx1* and *Bx8*, respectively (Ahmad *et al.*, 2011; Gierl and Frey, 2001), are located on chromosome 1. In response to insect feeding, DIMBOA-Glc is activated by glucosidases to form DIMBOA, which then decays to active-insect-deterrent metabolites (Gierl and Frey, 2001). Additionally, in response to chewing herbivores, DIMBOA-Glc is converted to the more toxic 2-hydroxy-4,7-dimethoxy-1,4-benzoxazin-3-one acid glucoside (HDMBOA-Glc) (Glauser *et al.*, 2011; Oikawa *et al.*, 2004), most likely by the Bx10 methyltransferases of maize (Meihls *et al.*, 2013).

*Rhopalosiphum maidis* (corn leaf aphid) is a pest on several economically important monocot crops, including maize, sorghum, wheat, barley, and *Miscanthus sinensis* (Carena and Glogoza, 2004; Huggett *et al.*, 1999). In the case of maize, all aboveground plant parts are susceptible to *R. maidis*. Infestation at the seedling stage slows development, reduces plant height, and decreases yield (Bing and Guthrie, 1991). Other damage occurs through tassel infestations, where the accumulation of sticky honeydew can prevent pollen shed, and yield losses of up to 90% have been reported (Carena and Glogoza, 2004; Foott and Timmins, 1973). Additionally, *R. maidis* can transmit damaging plant viruses, including *Maize dwarf mosaic virus* and *Barley yellow dwarf virus* (Saksena *et al.*, 1964; Thongmeearkom *et al.*, 1976).

There is long-standing evidence for natural variation in maize resistance to *R. maidis* (Awadallah and Hanna, 1985; Bing and Guthrie, 1991; Bing *et al.*, 1992; Chang and Brewbaker, 1976; Everly, 1960; Gemert, 1917; Huber and Stringfield, 1942; Long *et al.*, 1977; Lu, 1999; Lu and Brewbaker, 1999; McColloch, 1921; Meihls *et al.*, 2013; Rhodes and Luckmann, 1967; Snelling *et al.*, 1940; So, 2003; So *et al.*, 2010; Walter and Brunson, 1940). Whereas some studies indicate monogenic resistance (Chang and Brewbaker, 1976; Lu and Brewbaker, 1999), others show multiple genes with additive effects (Bing and Guthrie, 1991; Bing *et al.*, 1992; Long *et al.*, 1977). In one series of experiments, aphid resistance was recessive, and further analysis identified two resistance loci, *aph* on chromosome 10 and *aph2* on the short arm of chromosome 2, which are associated with this trait (Chang and Brewbaker, 1976; Lu, 1999; Lu and Brewbaker, 1999; So, 2003; So *et al.*, 2010).

Parental lines of the maize nested association mapping (NAM) population (McMullen *et al.*, 2009b; Yu *et al.*, 2008; www.panzea.org) vary greatly in their resistance to *R. maidis* (Meihls *et al.*, 2013). Genetic mapping of this trait using RILs derived from crosses between B73 and inbred lines that are more aphid-sensitive (CML 52, CML69, CML277, and CML322) identified a natural transposon insertion in *Bx10c* (GRMZM2G023325). This gene encodes a methyltransferase that converts DIMBOA-Glc to HDMBOA-Glc (Meihls *et al.*, 2013). Aphids produce more progeny on plants with a functional methyltransferase and constitutively high levels of HDMBOA-Glc. Maize lines with a transposon insertion in the methyltransferase gene have higher levels of DIMBOA, which, unlike HDMBOA-Glc, can induce callose accumulation and perhaps other maize defence responses (Ahmad *et al.*, 2011). Thus, even though HDMBOA-Glc is more toxic to aphids *in vitro* than DIMBOA-Glc, plants with higher levels of DIMBOA-Glc and DIMBOA are more resistant to *R. maidis* (Meihls *et al.*, 2013).

Whereas the *Bx10c* gene was identified by studying maize inbred lines that are more aphid-sensitive than the reference line, B73, Mo17 is one of the most aphid-resistant inbred lines among the 26 that were tested (Meihls *et al.*, 2013). As both B73 and Mo17 contain the same inactivating transposon insertion in the *Bx10c* gene (Meihls *et al.*, 2013), this locus would not affect aphid resistance in the well-characterized inter-mated B73 by Mo17 (IBM) RIL population (Lee *et al.*, 2002). Thus, it was hypothesized that genetic mapping with these RILs would identify novel maize aphid resistance QTL. Data presented here show that at least two major loci have additive effects on aphid resistance in inbred line Mo17 relative to B73.

## Materials and methods

### Plants and growth conditions

Maize plants for metabolite analysis and insect bioassays were grown in corn mix [produced by combining 0.16 m^3^ Metro-Mix 360 (Scotts, Marysville, OH, USA), 0.45kg finely ground lime, 0.45kg Peters Unimix (Scotts), 68kg Turface MVP (Profile Products, Buffalo Grove, IL, USA), 23kg coarse quartz sand, and 0.018 m^3^ steam-treated field soil]. Seeds were planted ~1.5cm deep in 8×8cm pots and were placed in Conviron (Conviron, Winnipeg, Canada) growth chambers. Growing conditions consisted of a 16:8h light:dark photoperiod, 180 μmol photons m^−2^ s^−1^ light intensity, 23 °C temperature, and 60% humidity. All experiments were conducted with two-week-old maize seedlings (V2–V3 growth stage).

### Aphid growth assays

*R. maidis* was obtained from Stewart Gray (USDA Plant Soil and Nutrition Lab, Ithaca, NY) and the colony was maintained on seedlings of maize inbred line B73. For genetic mapping,142 RILs derived from crosses between B73 and Mo17 (a subset of the maize IBM population; Lee *et al.*, 2002) were used for genetic mapping. Ten *R. maidis* aphids were confined on two-week-old plants with microperforated polypropylene bags (15 cm×61cm; PJP Marketplace, http://www.pjpmarketplace.com), and the progeny were counted 7 d later. Experiments with the 142 RILs were conducted in triplicate and the results were averaged for QTL analysis. For experiments to assess aphid growth on B73, Mo17, NILs, and W22 *Bx1::Ds* segregating mutant stocks, bioassays were conducted in a similar manner.

### Callose staining

Ten adult aphids were confined to a clip cage for 72h on the third leaf of two-week-old maize seedlings. Control plants received cages without aphids. Three days later, leaf material within the aphid cage was excised and used for callose staining as described previously (Luna *et al.*, 2011; Ton *et al.*, 2005). Leaves were de-stained in 98% ethanol for at least 48h until all tissue was transparent, washed in 0.07M phosphate buffer (pH 9), incubated (stained) for 2h in 0.07M pH 9 phosphate buffer containing 0.01% aniline-blue (Sigma, St. Louis, MO), and stored at 4 °C in 0.07M pH 9 phosphate buffer until microscopic analysis. Observations were performed with an Leica DM5500 epifluorescence microscope equipped with a ×20 immersion objective, a UV filter (BP 340 to 380nm, LP 425nm) a Retiga-2000R colour CCD camera, and Qcapture Pro 6.0 acquisition software (Leica, Wetzlar, Germany). Callose spots were quantified on the adaxial side of the leaf segment contained within the clip cage (~146mm^2^, with or without aphid feeding) and number of callose spots was calculated per mm^2^ of leaf tissue.

### Benzoxazinoid assays

Groups of ten aphids were confined to individual leaves of two-week-old maize plants, as described above for the callose assays. The maize leaf tissue within the cage was harvested, weighed, and extracted in 30% methanol:0.1% formic acid:69.9% deionized water (v:v:v). Samples of the extract were analysed by HPLC-absorbance detection using a C18 reverse-phase Luna column (5 μm pore size, 250×4.60mm; Phenomenex, Torrance, California, USA), a Waters 2695 pump system (Waters, Milford, MA, USA), and a Waters 2996 absorbance detector. The HPLC solvents were A, 0.1% v/v formic acid in deionized water, and B, 0.1% v/v formic acid in methanol, with a flow rate of 1ml min^–1^. The following gradient was used for the analytical analysis: 0–15min, gradient from 20%–30% B; 15–25min, gradient to 40% B, 25–30min, 40% B; and 35–40min, 20% B. Benzoxazinoid abundance was calculated from a standard curve that was produced with authentic standards that were kindly supplied by Gaetan Glauser (University of Neuchatel, Neuchatel, Switzerland).

### *Bx1::Ds* transposon insertion identification

A *Ds* transposon insertion (B.W06.0775) in exon 4 of the *Bx1* gene (GRMZM2G085381) in the W22 genetic background was identified through the *Ds* project website (http://acdstagging.org; Vollbrecht *et al.*, 2010). Seed stocks segregating this insertion were planted and primers were designed to identify plants carrying homo- or heterozygous *Bx1::Ds* alleles, using the B73 RefGen_v2 genome browser at MaizeGDB (www.maizegdb.org) in conjunction with Primer3 software (http://www-genome.wi.mit.edu/genome_software/other/primer3.html). DNA was extracted from seedling leaf tissue using a CTAB (cetyltrimethyl ammonium bromide)-based extraction protocol (adapted from Fulton *et al.*, 1995). A *Ds* 5’-end primer (GTTCGAAATCGATCGGGATA) was used in combination with a primer designed to the chromosomal sequence flanking the B.W06.0775 *Ds* insertion (TCTTA ACCTCCTGGATGAGTG). The insertion was confirmed using a 20 μl GoTaq PCR reaction (Promega, Fitchburg, Wisconsin) with 4% dimethylsulfoxide, using the following conditions: initial denaturation 94 °C for 3min; 35 cycles of 94 °C for 30 s, 57 °C for 30 s, 72 °C for 1min; and final extension 72 °C for 10min. Seedlings were genotyped in a second PCR assay to distinguish *Ds* insertion homozygotes from heterozygotes and wild-types. For this assay, a second *Ds* flanking primer complementary to chromosomal DNA on the other side of the *Ds* insertion site (GCCAAGAACAACAACCTGGAGC) was designed using the methods described above. The two *Ds*-flanking primers were used to amplify the wild-type allele in *Ds* heterozygotes and wild-type plants. The segregating population was subjected to benzoxazinoid analysis by HPLC, as described above. Seed stocks segregating the unstable B.W06.0775 homozygote (in an *Ac*-immobilized W22 genetic background; Conrad and Brutnell, 2005) are available from the Maize Genetics Cooperation Stock Center (http://maizecoop.cropsci.uiuc.edu/) as AcDs-00565.

### Data analysis

Windows QTL Cartographer (WinQTL Version 2.5, http://statgen.ncsu.edu/qtlcart/WQTLCart.htm) was used for composite interval mapping. Marker data for the B73×Mo17 RIL population were provided by Peter Balint-Kurti (Balint-Kurti *et al.*, 2007). The WinQTL program settings were: CIM program module=Model 6: Standard Model, walking speed=1 cM, control marker numbers=5, window size=10 cM, regression method=backward regression. A permutation procedure (Churchill and Doerge, 1994) was run 500 times to determine the *P*<0.05 LOD significance threshold. Other statistical tests were conducted using JMP software (www.jmp.com).

## Results

**Fig. 1.**
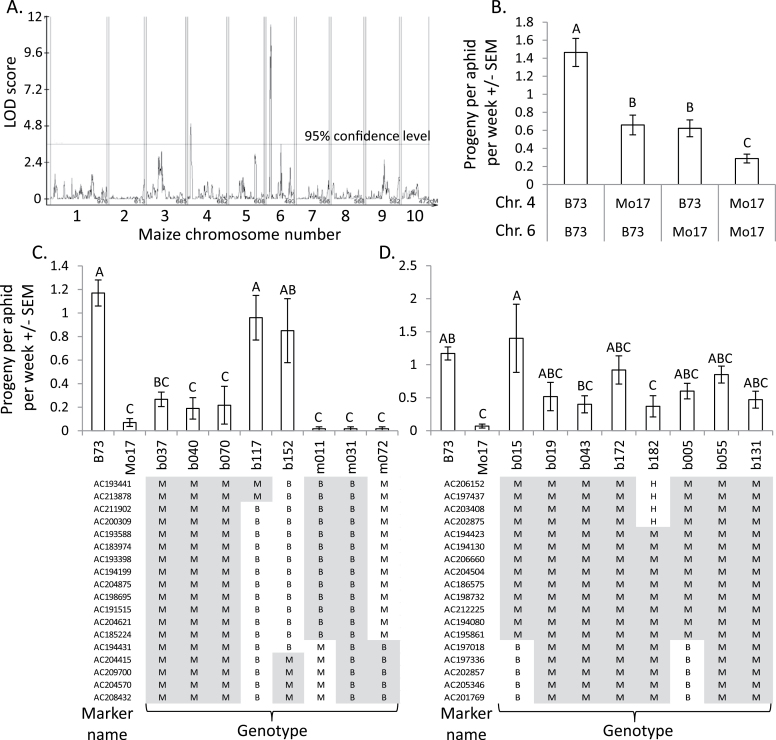
Mapping QTL for *R. maidis* resistance using B73×Mo17 recombinant inbred lines (RILs) and near-isogenic lines (NILs). (A) QTL for aphid progeny production were detected on chromosomes 4 and 6 by composite interval mapping. (B) Aphid progeny production on RILs that were separated according to whether they have the B73 or the Mo17 allele for the QTL on chromosomes 4 and 6, respectively. (C) Aphid reproduction on NILs with reciprocal insertions of the chromosome 4 QTL region into B73 and Mo17. Mean±SEM of *n*=6–10. (D) Aphid reproduction on NILs with insertions of the chromosome 6 QTL region of Mo17 into B73. Mean±SEM. of *n*=6–10. B=B73 genotype, M=Mo17 genotype, H=heterozygous, and shaded areas indicate the reciprocal introgressions into the other genetic background. Different letters above bars indicate significant differences, ANOVA followed by Tukey’s HSD.

Aphid nymph production was measured on 142 RILs of the maize IBM mapping population. Analysis of these data showed significant QTL on chromosomes 4 and 6 (Fig. 1A), accounting for 15% and 27% of the total variance in aphid resistance, respectively. QTL for the survival of adult aphids that were placed on the RILs were found at the identical positions on chromosomes 4 and 6 (data not shown). For both loci, the allele conferring higher aphid resistance came from the Mo17 parent in the cross. The effects of the two QTL are additive and aphid reproduction on RILs that have the Mo17 allele on both chromosomes 4 and 6 is significantly lower than on RILs that have only one or the other Mo17 allele (Fig. 1B).

B73×Mo17 NILs (Eichten *et al.*, 2011) were used to confirm the effects of the aphid resistance QTL on chromosomes 4 and 6. In experiments with NILs that have Mo17 segments of the chromosome 4 QTL region introgressed into B73, lines b037, b040, and b070 exhibited reduced aphid reproduction relative to B73, whereas lines b117 and b152 did not (Fig. 1C). This defined an interval between markers AC213878 and AC204415 (1.35 Mbp on chromosome 4; maize RefGen v2, www.maizegdb.org) as the position of the Mo17 aphid resistance locus. In contrast, aphid reproduction on NILs with introgressions of the B73 allele into the Mo17 genetic background (lines m011, m031, and m072) was similar to reproduction on Mo17 (Fig. 1C), suggesting that there are additional aphid resistance loci acting in the overall Mo17 genetic background.

In the case of the chromosome 6 QTL, only introgressions of the Mo17 allele into B73 were available. These lines showed an intermediate aphid reproduction phenotype (Fig. 1D) and, on average, aphid reproduction on lines with a segment of Mo17 chromosome 6 introgressed into B73 was approximately 0.5 nymphs per week lower of that on inbred line B73 (0.67±0.09 vs. 1.17±0.13 progeny per week; *P*<0.05, *t-*test). The effect of this introgression is similar to the magnitude of the chromosome 6 effect on aphid resistance that was observed in the QTL mapping (Fig. 1B).

**Fig. 2.**
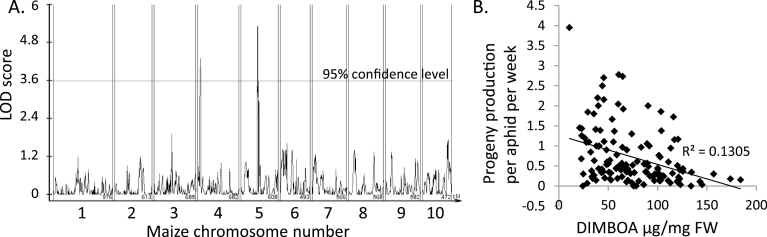
DIMBOA content in B73×Mo17 RILs. (A) Location of QTL for DIMBOA content on maize chromosomes 4 and 5 using composite interval mapping and B73×Mo17 RILs. (B) Correlation of DIMBOA content and aphid reproduction on B73×Mo17 RILs. A best-fit line was placed by linear regression, r=–0.361, *P*<0.05, Pearson correlation.

The chromosome 4 aphid resistance QTL coincided with a region of the maize genome that contains at least eight genes of the benzoxazinoid biosynthesis pathway (Gierl and Frey, 2001). To determine whether benzoxazinoid content can influence *R. maidis* reproduction, DIMBOA abundance was mapped as a quantitative trait using 128 IBM RILs, a subset of the 142 that were used for aphid resistance mapping. This identified significant QTL on chromosomes 4 and 5, accounting for 11% and 8% of the variance in this trait, respectively (Fig. 2A). In both cases, the high-DIMBOA allele came from the Mo17 parent in the cross. QTL for DIMBOA-Glc abundance, which is highly correlated with DIMBOA abundance, are located at the same two chromosomal positions (data not shown). Aphid reproduction on the IBM population RILs was negatively correlated with DIMBOA content (*r=*–0.361, *P*<0.01, Pearson correlation; Fig. 2B). Previous research has shown that *R. maidis* feeding has no significant effect on B73 benzoxazinoid content (Meihls *et al.*, 2013). Similarly, in the current study, no significant changes were observed in the abundance of DIMBOA, DIMBOA-Glc, or HDMBOA-Glc in Mo17 response to aphid feeding (*P*>0.05, *t-*test).

**Fig. 3.**
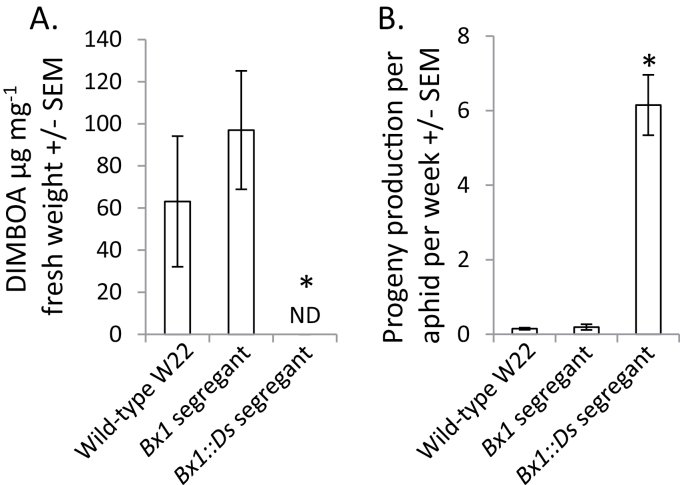
DIMBOA content of and *R. maidis* reproduction on a W22 *Bx1::Ds* knockout mutant. (A) DIMBOA content in a segregating *Bx1*::*Ds* transposon insertion line relative to wild-type W22. (B) *R. maidis* reproduction on maize inbred line W22 and a segregating *Bx1*::*Ds* transposon insertion line. *n*=4 (wild-type W22), 12 (*Bx1* segregants), and 8 (*Bx1::Ds* segregants). *P<0.05, Dunnett’s test relative to wild-type W22. ND=not detected.

Both an aphid resistance QTL (Fig. 1A) and a high-DIMBOA QTL (Fig. 2A) were mapped to a region of chromosome 4 containing DIMBOA biosynthetic genes. Association mapping with maize inbred lines linked DIMBOA abundance to genetic variation in *Bx1,* the first gene in the pathway (Butrón *et al.*, 2010), suggesting that *Bx1* by itself could affect aphid resistance. To test this hypothesis, a *Ds* transposon knockout mutation of *Bx1* was identified in the W22 maize genetic background. Homozygous *Bx1::Ds* progeny, confirmed by PCR-based genotyping, had no detectable DIMBOA (Fig. 3A) and aphid reproduction was significantly increased relative to wild-type W22 (Fig. 3B). In contrast, segregating wild-type siblings of the *Bx1::Ds* plants were not significantly different from wild-type W22 in either their DIMBOA content or their aphid resistance.

**Fig. 4.**
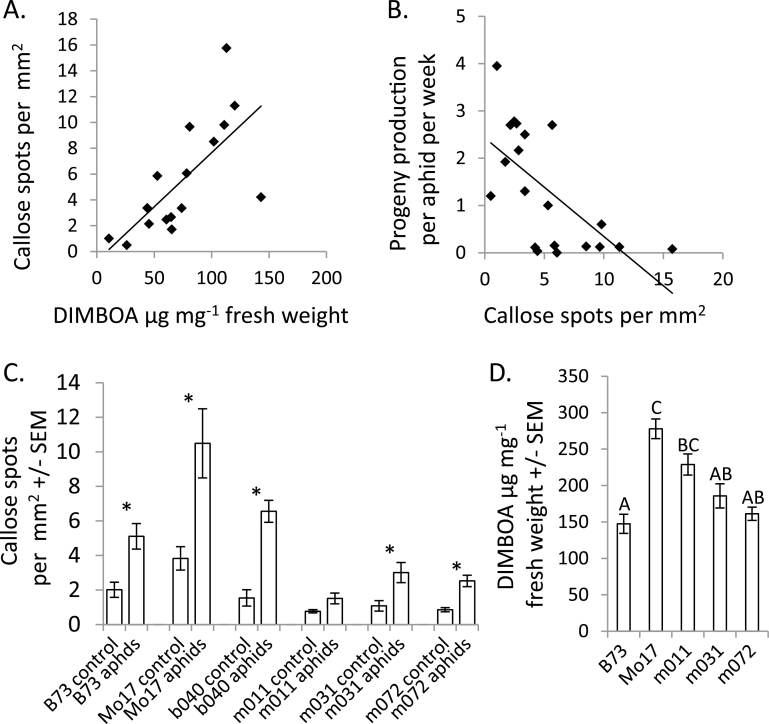
Correlation of callose accumulation and *R. maidis* reproduction. (A) Callose accumulation in response to aphid feeding on B73×Mo17 RILs is positively correlated with DIMBOA content. A best-fit line was placed by linear regression, r=0.693, *P*<0.05, Pearson correlation. (B) Aphid reproduction is negatively correlated with callose accumulation in B73×Mo17 RILs. A best-fit line was placed by linear regression, r=0.649, *P*<0.05, Pearson correlation. (C) Callose accumulation in B73, Mo17, and selected chromosome 4 QTL NILs. Mean±SEM,*P<0.05, *t-*test comparing aphid-fed and control plants. (D) Benzoxazinoid content in B73, Mo17, and NILs with the chromosome 4 QTL allele of B73 introgressed into Mo17. Different letters indicate significant differences, ANOVA followed by Tukey’s HSD test.

Previous research demonstrated that DIMBOA is required for callose induction in maize (Ahmad *et al.*, 2011) and that *R. maidis* resistance is correlated with both DIMBOA content and callose formation (Meihls *et al.*, 2013).To determine whether this trait influences aphid resistance in the IBM population, callose production was measured in the ten most aphid-resistant and the ten most aphid-sensitive RILs that were identified in the analysis shown in Fig. 1A. On average, the aphid-resistant RILs had three times as many callose spots in response to aphid feeding as the aphid-sensitive RILs (8.1±1.2 vs. 2.6±0.5 spots per mm^2^, *P*<0.01, *t-*test). Callose content in response to aphid feeding was positively correlated with DIMBOA content (*r=*0.695, *P*<0.01, Pearson correlation; Fig. 4B), consistent with prior reports that DIMBOA is required for aphid-induced callose formation (Ahmad *et al.*, 2011; Meihls *et al.*, 2013). Aphid reproduction was negatively correlated with callose accumulation (*r=*–0.649, *P*<0.01, Pearson correlation; Fig. 4C), an indication that elevated callose accumulation is part of a maize defence response against aphids.

Aphid-induced callose formation was measured in B73, Mo17, and selected NILs to confirm that aphid resistance associated with the chromosome 4 QTL (Fig. 1A) is associated with callose formation. Consistent with the lower aphid progeny production on Mo17 (Fig. 1), there was elevated callose production in this line relative to B73 (*P*<0.05, *t-*test; Fig. 4C). NIL b040, which has an introgression of the Mo17 allele of the chromosome 4 QTL into B73, has an intermediate level of callose production.

On average, NILs m011, m031, and m072, which have introgressions of the B73 QTL on chromosome 4 into M017, exhibit significantly lower levels of aphid-induced callose production than Mo17 (343±49 vs. 1532±292 spots per mm^2^, *P*<0.05, *t-*test; Fig. 4C). Benzoxazinoid assays were conducted to determine whether this reduced callose production is associated with lower DIMBOA accumulation owing to the introgression of the B73 *Bx* chromosomal region into Mo17 in these NILs. Two of the NILs (m031 and m072) had a DIMBOA content that was significantly lower than that of Mo17 and similar to that of B73. Despite the low DIMBOA content and callose formation, *R. maidis* reproduce quite poorly on m011, m031 and m072 (Fig. 1C). This observation is consistent with the hypothesis that higher aphid resistance in these three NILs relative to B73 is independent of DIMBOA-induced callose production associated with the chromosome 4 QTL (which is derived from B73 in the NILs), and is instead controlled by other loci in the overall Mo17 background of the NILs.

## Discussion

**Fig. 5.**
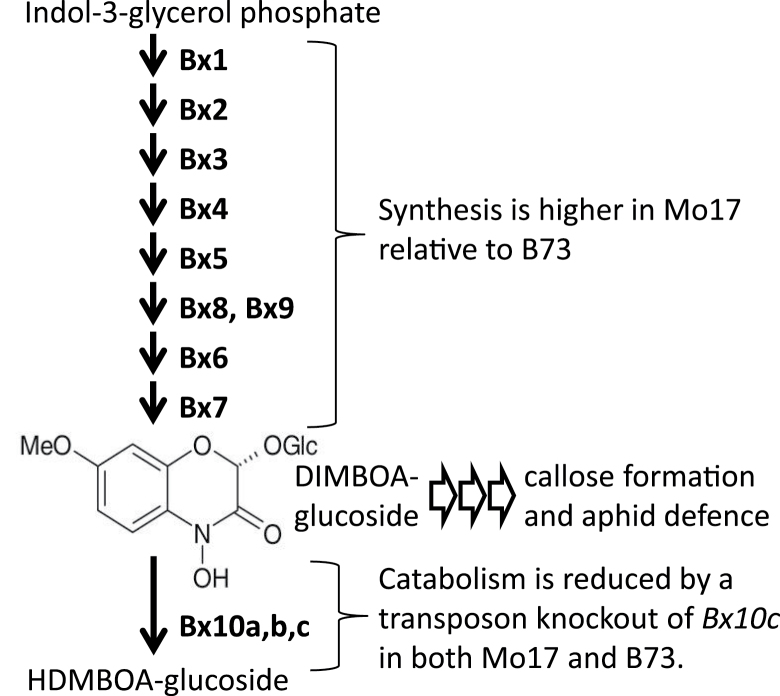
Model of aphid defence regulation by benzoxazinoid metabolism in maize seedlings. Both synthesis and catabolism regulate the abundance of DIMBOA-glucoside. Genetic variation in the biosynthetic pathway affects DIMBOA-glucoside abundance (Butrón *et al.*, 2010), and both Mo17 and B73 have a transposon insertion in *Bx10c*, which encodes a constitutively expressed DIMBOA-Glc catabolic enzyme (Meihls *et al.*, 2013). DIMBOA-Glc is a precursor for DIMBOA, which is required to trigger callose formation and perhaps other aphid defence responses in maize.

Results presented here, together with previous findings (Meihls *et al.*, 2013), show that both synthesis and catabolism of DIMBOA-glucoside contribute to aphid resistance in maize (Fig. 5). DIMBOA content of maize seedlings, which is highly correlated with the abundance of the precursor DIMBOA-glucoside, is an essential contributing factor for callose formation and *R. maidis* resistance (Ahmad *et al.*, 2011; Meihls *et al.*, 2013). Maize lines with a functional *Bx10c* gene (e.g. CML52, CML69, CML277, and CML322) have constitutive DIMBOA-Glc catabolism to HDMBOA-Glc and are aphid-sensitive. Both B73 and Mo17 contain a *Doppia*-like transposon insertion that inactivates *Bx10c* and thereby confers higher aphid resistance. In the case of Mo17, elevated biosynthesis further increases DIMBOA abundance and thereby causes this line to be even more aphid-resistant than B73 (Fig. 5). Fine-scale mapping of a DIMBOA QTL in the maize IBM population to a specific *cis-*regulatory region more than 100 kbp upstream the *Bx1* gene, together with the longer persistence of *Bx1* gene expression in developing Mo17 seedlings (Zheng *et al*., unpublished results), suggests this as a likely cause of elevated DIMBOA content, callose accumulation, and aphid resistance in Mo17 relative to B73.

Chromosome 5 also contains a QTL with a significant effect on DIMBOA abundance (Fig. 2A). However, unlike the chromosome 4 DIMBOA QTL, this was not associated with a QTL affecting aphid resistance (Fig. 1A). As the chromosome 5 QTL does not include any known benzoxazinoid biosynthesis genes, it may be a regulatory locus affecting the benzoxazinoid pathway and perhaps other maize defence responses. Thus, the absence of a significant aphid resistance QTL in this region could be explained by opposing effects of as yet unknown maize defence responses that are regulated by either by the chromosome 5 benzoxazinoid QTL or perhaps by nearby loci on chromosome 5 that were not resolved using the current genetic mapping approach.

Whereas introgression of the Mo17 chromosome 4 QTL into B73 decreased aphid reproduction (NILs b037, b040, and b070), the converse introgression (NILs m011, m031, and m072) did not have the opposite effect (Fig. 1C). Aphid resistance associated with the Mo17 genome seems to override any benefits that the B73 chromosome 4 QTL allele provides for aphid reproduction. Given the relatively low aphid-induced callose formation (Fig. 4C) and low DIMBOA content (Fig. 4D) in NILs m031, and m072, the observed aphid resistance that is provided by the Mo17 contribution to these NILs is likely to be independent of benzoxazinoid biosynthesis.

Consistent with the hypothesis of DIMBOA-independent aphid resistance mechanisms in Mo17, the genetic mapping interval of the chromosome 6 aphid resistance QTL (Fig. 1A) contains neither known benzoxazinoid biosynthesis genes, nor genetic evidence for a DIMBOA-related QTL (Fig. 2A). Thus, it is likely that the observed aphid resistance associated with Mo17 chromosome 6 involves something other than DIMBOA accumulation. The significant, but relatively weak correlation between DIMBOA concentration and aphid reproduction observed in experiments with B73×Mo17 RILs (Fig. 2B) also is consistent with the hypothesis of additional resistance mechanisms acting in Mo17. At this point, however, we cannot rule out the possibility that as yet unknown benzoxazinoid modifications that affect aphid resistance are influenced by the chromosome 6 QTL.

As benzoxazinoids are deleterious to a large variety of insect herbivores (Niemeyer, 2009), natural variation in *Bx1* and/or other benzoxazinoid biosynthesis genes in maize bin 4.01 probably plays a key role in maize defence, not only against *R. maidis* but also other insect herbivores. Similar to *R. maidis*, *Rhopalosiphum padi* (birdcherry-oat aphid) produced more progeny on a *bx1* mutant than on near-isogenic wild-type *Bx1* maize (Ahmad *et al.*, 2011). A QTL for maize DIMBOA accumulation was previously mapped to bin 4.01 using several RIL sets (Butrón *et al.*, 2010), and association mapping indicated that *Bx1* polymorphisms were the most likely cause of the phenotypic variation. QTL for the amount of damage caused by two lepidopteran herbivores, *O. nubilalis* and *O. furnacalis* (Cardinal *et al.*, 2006; Jampatong *et al.*, 2002; Krakowsky *et al.*, 2004; Xia *et al.*, 2010), also are located in bin 4.01 and may be related to natural variation in benzoxazinoid content. Specialist herbivores, such as *Diabrotica virgifera* and *Spodoptera frugiperda*, can be insensitive to variation in benzoxazinoid content (Davis *et al.*, 2000) or have specific DIMBOA detoxification enzymes (Glauser *et al.*, 2011). Thus, these insects may be insensitive to benzoxazinoid abundance or might even be attracted to plants with higher benzoxazinoid content.

The chromosomal locations of the two *R. maidis* resistance QTL described here (Fig. 1A) are different from those that have been mapped in previous studies (Meihls *et al.*, 2013; So *et al.*, 2010). The chromosome 6 QTL is particularly interesting, as further genetic mapping may lead to the discovery of novel aphid resistance mechanisms in maize. By identifying the underlying genetic basis of this *R. maidis* resistance QTL and others that may be found in future genetic mapping studies, it will be possible to develop new tools for improving the insect resistance of commercial maize lines through breeding or transgenic approaches.
